# Rapid progression of condyloma acuminatum caused by IL-17A antibody treatment: a case report

**DOI:** 10.3389/fmed.2024.1387620

**Published:** 2024-05-15

**Authors:** Fang Sun, Zhenze Yu

**Affiliations:** Department of Dermatology, Affiliated AoYang Hospital of Jiangsu University, Zhangjiagang, China

**Keywords:** psoriasis, condyloma acuminatum, secukinumab, HPV, IL-17A antagonist

## Abstract

Anti interleukin (IL)-17A therapy is a common and effective treatment for psoriasis, but there are also risks of infection. In this case, we presented a patient who experienced a swift progression of condyloma acuminatum on the genitals during psoriasis treatment with secukinumab, a human IL-17A antagonist. Through this case, we strongly suspect that anti IL-17A treatment may promote the onset and rapid progression of low-risk HPV-associated condyloma acuminatum.

## Introduction

Psoriasis, a chronic inflammatory skin condition, is associated with genetic susceptibility, environmental triggers, and immune dysregulation (1). In recent years, novel treatment methods targeting key immune molecules in the progression of psoriasis have emerged and proven to be highly effective (1). Secukinumab, a human interleukin (IL)-17A antagonist, is widely used in the clinical treatment of plaque psoriasis. However, considering the significant role of IL-17A in anti-infection, the use of IL-17A antagonists may also carry increased risks (2). Mucocutaneous candidiasis and upper respiratory tract infections have been reported in patients treated with IL-17A inhibitors (3). In this case, we presented a patient who experienced a swift progression of condyloma acuminatum on the genitals during psoriasis treatment with secukinumab.

## Case presentation

**Figure 1:**
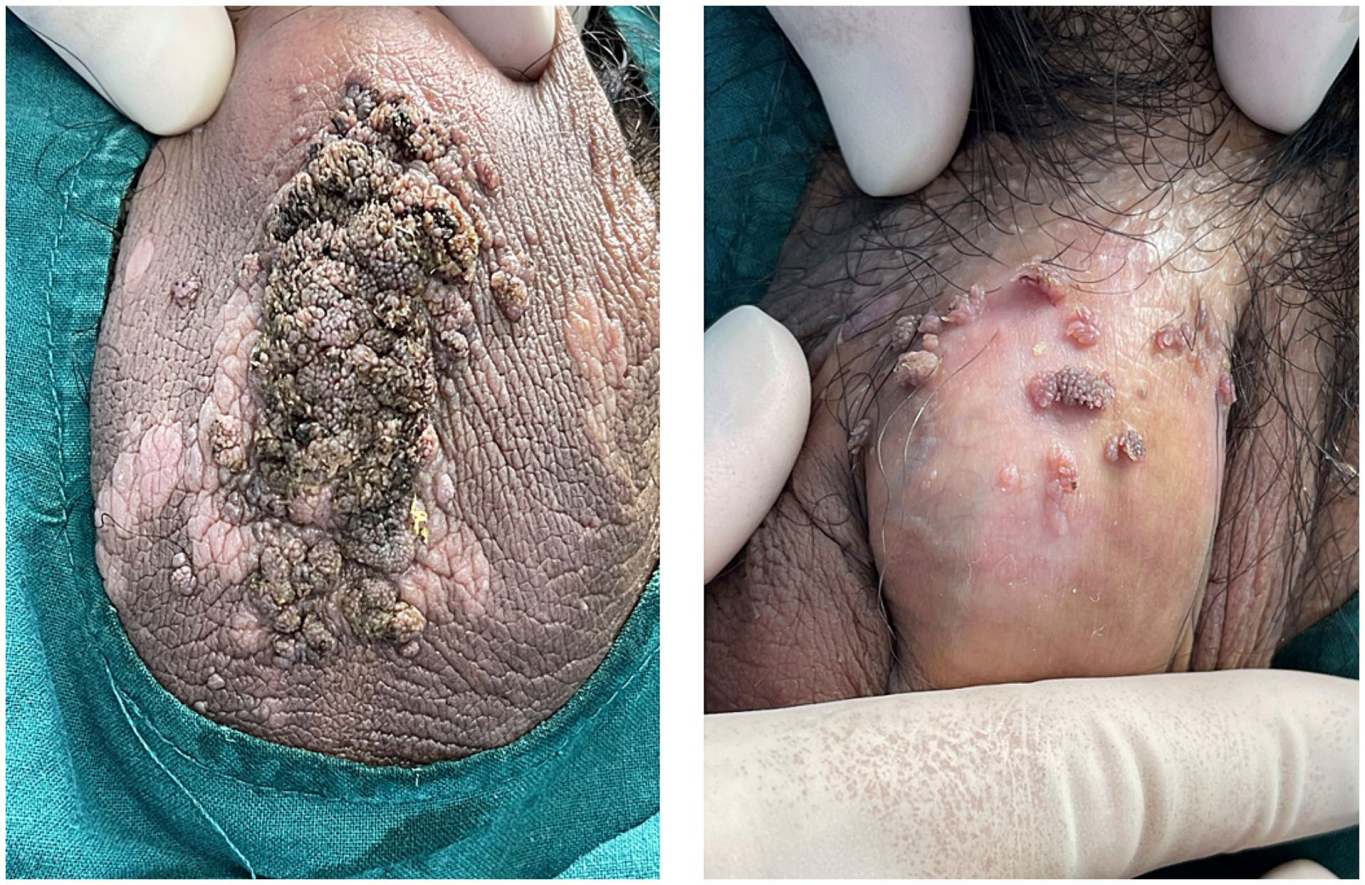
Typical skin lesions on the scrotum and penis of the patient.

**Figure 2:**
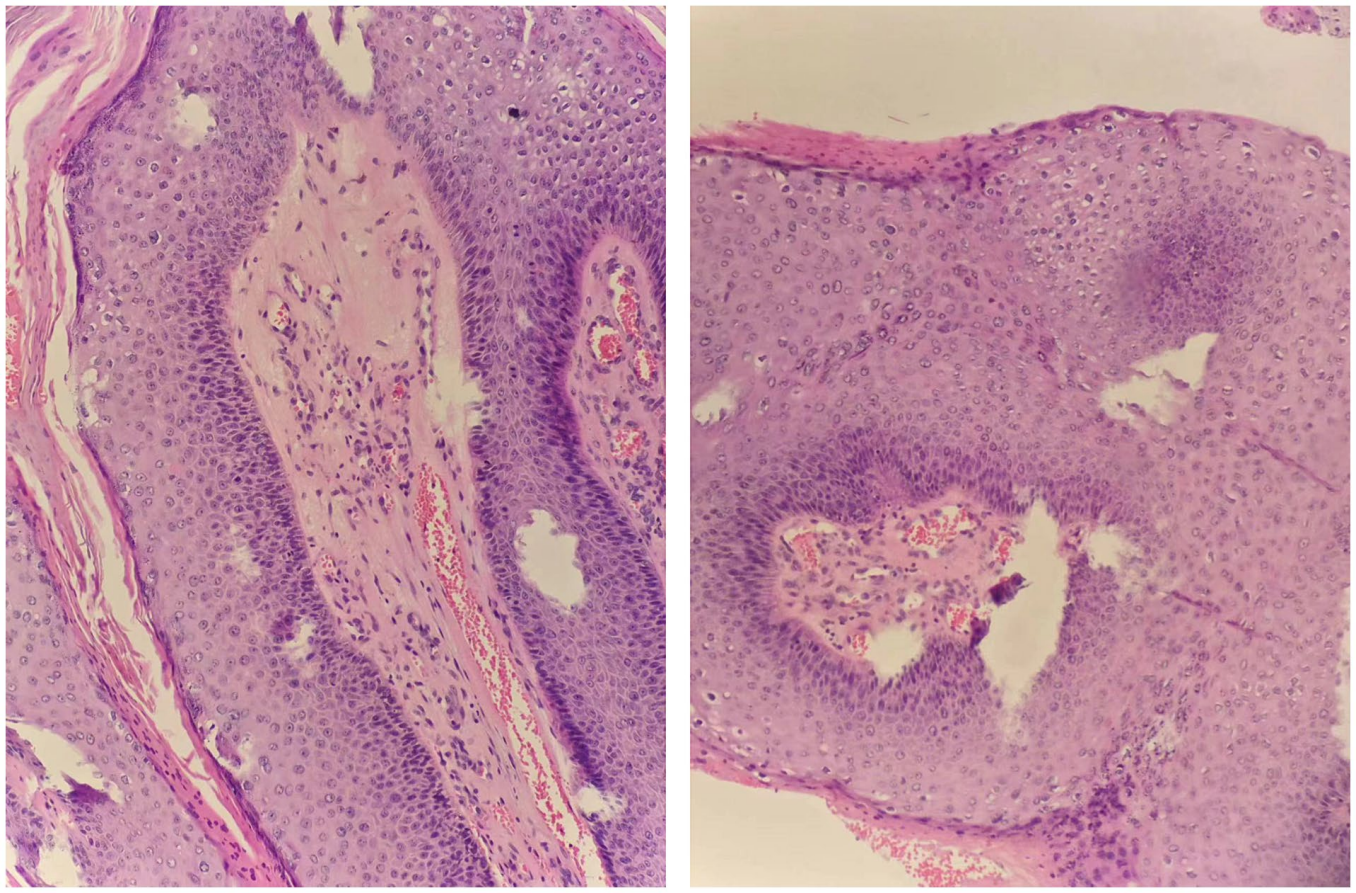
Pathological examination of scrotal and penile skin lesions (HE staining, 40×).

**Figure 3:**
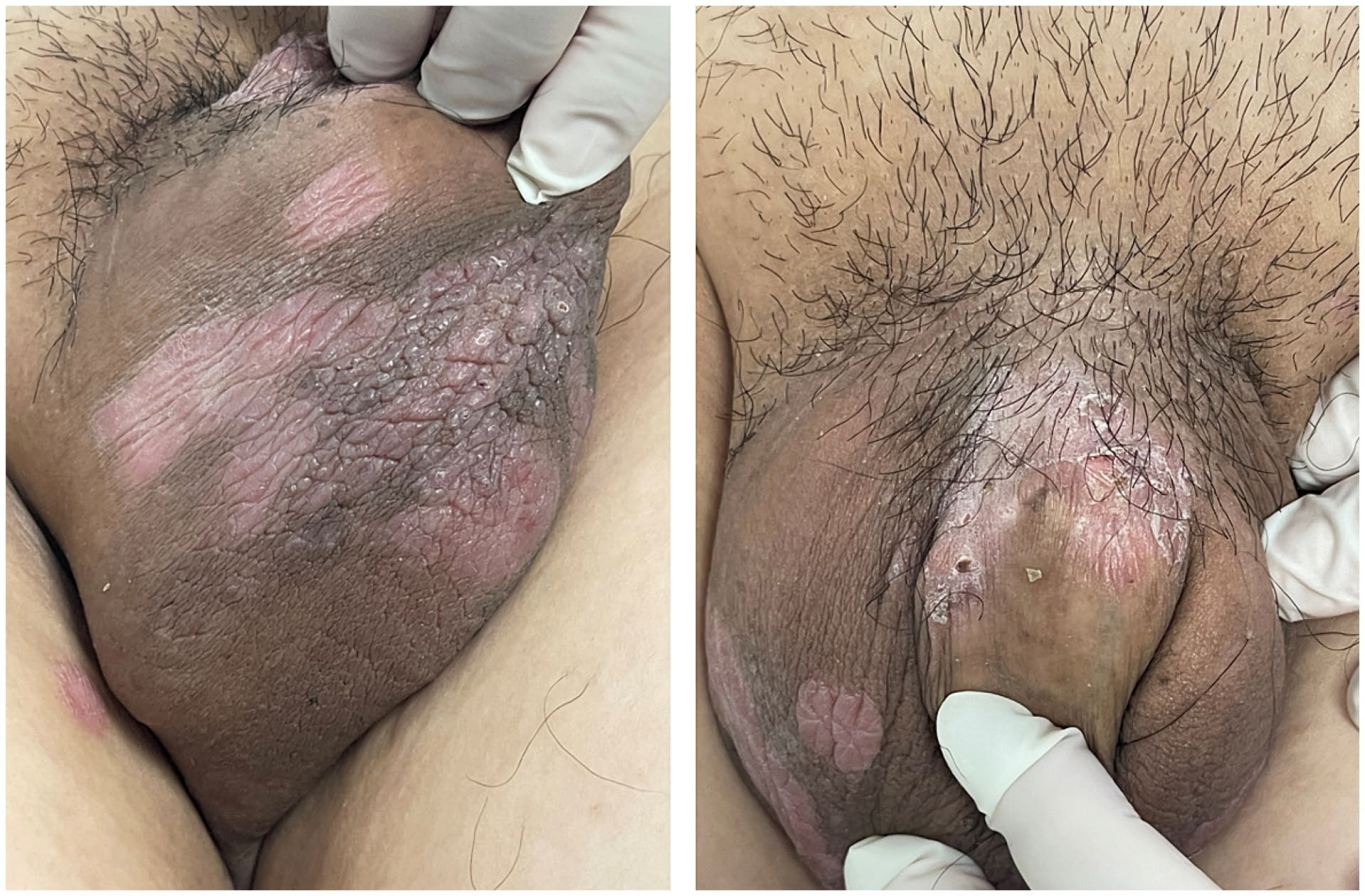
No condyloma acuminatum relapsed at the original skin lesion sites at the patient’s last follow-up.

A 33-year-old male presented to us with a 10-year history of plaque psoriasis which worsened 15 months ago. Lesions appeared on the head, trunk, limbs, and scrotum. He was initially treated with oral Acitretin capsules 30 mg/d and topical clobetasol propionate cream for 1 month, but the effect was unsatisfactory, and the skin lesions continued to worsen. After excluding contraindications such as malignant tumors and infection, the patient was treated with secukinumab. Nine months ago, multiple sesame seed-sized brown papules appeared on the original skin lesion of the scrotum, which were initially asymptomatic. Within 1 month, the skin lesions rapidly enlarged and merged into patches, and similar lesions also appeared on the penis, acquiring a wart-like appearance and emitting a foul odor. Physical examination showed a brown patch of approximately 6.0 × 4.0 × 0.5 cm on the scrotum, consisting of numerous pedunculated vegetations with a wart-like appearance, and multiple similar lesions could be seen on the penis. Multiple pale red patches were also present on the scrotum, with a few scales on the surface (Figure 1). HPV-DNA examination confirmed the presence of HPV11, and serological examination showed no HIV infection. Pathology results revealed excessive keratinization and incomplete keratinization, hypertrophic spinous layers, and papillomatous hyperplasia, consistent with the diagnosis of condyloma acuminatum; the tumor cells show no atypia and they do not exhibit invasive growth, thus ruling out the possibility of Buschke-Löwenstein tumor (Figure 2). This patient was finally diagnosed with condyloma acuminatum. Considering that the patient has been treated with secukinumab and the condyloma acuminatum rapidly increase within one month, we strongly suspect that the onset and progression of condyloma acuminatum are related to the use of secukinumab. Considering the large size of the skin lesions and the patient’s own need for rapid removal of the lesions, we perform surgery to gradually remove the skin lesions using cutting and electrocautery techniques. The patient continued to receive secukinumab after sugery. During the 8-month follow-up period, there was no recurrence of condyloma acuminatum (Figure 3). The patient is very satisfied with our treatment.

## Discussion

In this case, the patient has psoriasis in the genital area, a site of disease that is underrecognized and undertreated. Most systemic treatments for psoriasis may benefit patients with genital involvement, including secukinumab (4). Secukinumab treatment is effective in improving skin lesions in the scrotum of this patient.

From an immunological perspective, there is a complex and close relationship between psoriasis and viral infection. Psoriasis is an chronic inflammatory skin disease in which IL-17 and IL-23 are key drivers of disease pathogenesis. Viral infection can act as a triggering factor, activating immune cells such as T lymphocytes and dendritic cells, promoting the expression of various inflammatory cytokines, and inducing or exacerbating psoriasis (5). Additionally, skin barrier damage caused by psoriasis can increase the risk of viral infection. In this case, condyloma acuminatum partially developed at the site of psoriasis skin lesions (5). On the other hand, immune cell infiltration and inflammatory cytokines produced during psoriasis pathogenesis have antiviral effects and promote antiviral immunity (5).

Previous studies have shown an increased risk of psoriasis after HPV infection, possibly due to the stimulation of systemic interleukin-17 (IL-17) production following HPV infection (6). The impact of IL-17A monoclonal antibody treatment on HPV infection in psoriasis patients is still unclear and controversial. Some research suggests that the HPV infection rate is significantly increased in psoriasis patients treated with IL-17 monoclonal antibodies (7), while other reports indicate therapeutic effects on HPV infection (5). Specifically, when using secukinumab to treat psoriasis, there is a significant decrease in HPV virus detection and even disappearance of condyloma acuminatum in some patients (8, 9), especially for high-risk HPV types (9). In this case, the patient was infected with HPV11, which is a low-risk type, indicating a potential difference in efficacy between IL-17A monoclonal antibodies against high-risk and low-risk HPV viruses. Additionally, the application of secukinumab led to a rapid increase in condyloma acuminatum in this patient, highlighting the impact of IL-17A monoclonal antibody treatment on the progression of condyloma acuminatum. These findings provide innovative insights as they differ from previous reports on the incidence of HPV-related diseases.

## Conclusion

In conclusion, this case suggests that IL-17A monoclonal antibody treatment may promote the onset and rapid progression of low-risk HPV-associated condyloma acuminatum. However, this study is a single case report with limitations, and further cases are needed to validate our findings.

## Data availability statement

The original contributions presented in the study are included in the article/supplementary material, further inquiries can be directed to the corresponding author.

## Ethics statement

As this is a single case report, ethics committee approval is not required. The patient in this manuscript has given written informed consent to publication of his case details and images.

## Author contributions

FS: Conceptualization, Data curation, Formal analysis, Investigation, Resources, Validation, Visualization, Writing – original draft. ZY: Validation, Writing – review & editing.
